# Macro-level mental health system indicators and cross-national suicide rates

**DOI:** 10.1080/16549716.2020.1839999

**Published:** 2021-01-19

**Authors:** Johnny Andoh-Arthur, Samuel Adjorlolo

**Affiliations:** a Department of Mental Health, School of Nursing and Midwifery, College of Health Sciences, University of Ghana, Accra, Ghana; b Research and Grant Institute of Ghana, Accra, Ghana; c Department of Psychology, School of Social Sciences, University of Ghana, Accra, Ghana

**Keywords:** Suicide, mental health, mental health governance, mental health professionals, suicide prevention

## Abstract

**Background:**

The relationship between macro-level mental health system indicators and population suicide rates is an area of contention in the literature, necessitating an analysis of current cross-national data to document any new trend in the relationship.

**Objective:**

This study investigated whether mental health system indicators are associated with national suicide rates.

**Method:**

Using an ecological study design and multivariate non-parametric robust regression models, data on suicide rates and mental health system indicators of 191 countries retrieved from WHOs 2017 Mental Health Atlas were compared.

**Results:**

Findings revealed that the average suicide mortality rate was significantly higher in high- income countries, relative to low-income countries. High-income countries are significantly more likely to have high number of mental health professionals, mental health policies and legislation, independent mental health authority and suicide prevention programs. These mental health system indicators demonstrated significant and positive association with suicide, suggesting that countries scoring high on these factors have higher odds of being categorized as high suicide risk countries.

**Conclusion:**

The findings have several implications for policy and practice, including the need to make existing mental health systems very responsive to suicide prevention.

## Background

Suicide accounts for about 800,000 deaths annually at an estimated age standardized rate of 11.2 per 100,000 [1]. Across the globe, suicide is the 15th most common cause of death [1,2]. Suicide also reportedly accounted for 1.4% of premature deaths worldwide in 2015 [3]. A recent study revealed that the age standardized mortality rate for suicide has decreased by 32.7% worldwide between 1990 and 2016, however, total number of deaths from suicide within the same period increased by 6.7% globally over the 27-year-study period [4]. Suicide is notably a complex, multifactorial phenomenon that shows significant variations, particularly with respect to the causal factors and mechanisms, as well as prevention and management strategies [5].

Mental health problems such as mood, substance use, psychotic or personality disorders have been shown to play critical roles in suicide trajectory [6–11]. Often repeated are some psychological autopsy studies from High-Income Countries that for instance, suggest that 90%–95% of individuals who die of suicide had a diagnosable psychiatric disorders at the time of committing suicide [7,8,12]. Some authors have challenged this statistic by pointing either to methodological flaws that usually produces the statistic or to evidence suggesting no diagnosable psychiatric disorders in some suicides in other contexts [13,14]. Indeed a systematic review from the Low-and Middle-Income countries (LMICs) has pointed to crucial role of non-psychiatric factors such as poverty in suicides [15]. It is possible that where mental health service is organized almost exclusively to focus on biomedical factors, it is most likely that less priority will be given to non-psychiatric factors in suicides [16]. Impliedly, cross-national variations in the role of psychiatric disorders and for that matter suicide rates generally could be partly attributed to variations in patterns, frequency, and meanings of suicide, as well as variations in the organization and provision of mental health service [6,16–18]. Among the factors that are likely to influence suicide rates across regions and countries are national income, mental health governance system, and resources for mental health, the so-called macro-level indictors of suicide because they require political commitment [19–21].

A number of ecological, country-level studies have examined the presumed link between suicide rate and mental health system indicators, such as availability of mental health services [5], professional density [2,22,23], mental health spending [24], mental health legislations and policies [21] and antidepressant sales [23]. A study conducted in Finland found that the prominence of outpatient services was associated with lower suicide rate [17] whereas others have reported high suicide rates in countries with better mental health services such as higher numbers of psychiatric beds and availability of training in mental health for primary care professionals [5,18]. With respect to professional density and suicide rate, findings are mixed, with some studies reporting negative relationship [2,23,25,26], positive relationship [5,18,22], or no statistically significant relationship [23]. An Austrian study, for instance, found that a high number of mental health professionals was associated with lower rates of suicide, adjusting for per capita alcohol consumption and unemployment rates [23]. In contrast, cross-national data from 191 countries revealed that the number of psychiatrists was significantly positively associated with population suicide rates [5]. In yet another study from Austria, no statistically significant relationship was observed between psychiatrist density and suicide rate [23].

Analyses of cross-national data showed an increase in suicide rates and existence of mental health initiatives such as mental health policy, mental health program [18,21]. More importantly, while suicide rates reportedly differ across countries [6], the direction of the difference is an area of contention. More specifically, contrary to the finding that a vast majority, namely 78%, of suicides occur in the Low and Middle-Income Countries [3], others have found a high prevalence of suicide in high-income countries, relative to LMICs [27,28]. In their analysis of cross-national data [18], found that countries with high mental health expenditure tend to experience high suicide rate.

The inconsistent findings provide impetus for more studies to examine suicide and mental health systems. There is the pressing need to subject current cross-national data to empirical analysis to document any new trend in the relationship between mental health system and suicide rates across geographical regions [5,22]. Consequently, this study is designed to first and foremost investigate the distribution of suicide rates across countries based on their national income, and second adopt an ecological framework^1^ ^1^...........and second adopt an ecological framework to investigate the associations between suicide rate and mental health systems indicators (e.g., the number of professionals, availability of mental health policies). Ecological framework views problems i.e. suicide as the outcome of interaction among many factors at multiple levels – the individual, the relationship, the community, and the societal. to investigate the associations between suicide rate and mental health systems indicators (e.g. the number of professionals, availability of mental health policies)..

## Methods

### Data source

The data were extracted from the mental health Atlas 2017 published by the WHO for member states. The Atlas is designed to monitor and evaluate progress made by member countries in achieving the WHO’s Comprehensive Mental Health Action Plan by 2020. Among the core requirements of the Action Plan are for member countries to, for instance, strengthen mental health governance, render comprehensive and integrated mental health care, and institute mental health promotion and prevention strategies [29]. The Atlas, therefore, provides information on the state of mental health across nations by unearthing the existence or otherwise of mental health-specific indices pertinent to the Comprehensive Mental Health Action Plan. These include policies, plans and laws for mental health, human and financial resources; the type of mental health facilities, and availability of mental health promotion and prevention programs.

### Data collection procedure

As noted previously, the Atlas is produced by the WHO with data from member countries. For the 2017 Atlas, data were collected using a structured questionnaire from 177 out of the 194 WHO member states, representing 97% of the world’s population. The questionnaires were developed in consultation with member states and internationals experts in the area of mental health care measurement. In each member state, a focal person was identified to complete the questionnaire by extracting data from multiple sources, including from local team of experts, institutions such as Mental Health Authority/Commission and psychiatric and non-psychiatric hospitals located across different parts of the country. Once the focal person has completed the data-gathering process, the questionnaires were sent back to the WHO for processing and use. Where applicable, the focal persons were re-contacted for further information and clarifications relating to the questionnaire to ensure data quality. Detailed procedure for data collection has been described elsewhere [30].

### Measures/instrument

#### Dependent variable

##### Suicide

The suicide mortality rate for the various countries were derived from the World Health Statistics data visualization dashboard, which is available at http://apps.who.int/gho/data/

#### Independent variables

The 2017 Atlas contain different macro-level data on mental health service delivery. Some variables (e.g. government’s total expenditure on mental health, number of mental health hospitals, and number of psychiatric units in a general hospital) were missing data at a scale that does not allow meaningful comparison. For example, in some instances, data are available for high-income countries but not of other income groups. Consequently, a decision was reached to exclude some data from the analysis. Variables with complete data across the various income groups were considered in this study, as stated below;

##### Income group

The 2017 Atlas categorized WHO member countries into four different income groups using the gross national income (GNI) developed by the World Bank in 2016. These are low income (GNI per capita of US$ 1,025 or less), low-middle income (GNI per capita between US$ 1,026 and US$ 4,035), upper-middle income (GNI per capita between US$ 4,036 and US$ 12,475), and high income (GNI per capita of US$ 12,476 or more).

##### Mental health system governance

The following variables were extracted to index mental health system governance: (1) Stand-alone policy or plan for mental health; (2) plan or strategy for child and/or adolescent mental health; (3) Stand-alone law for mental health; (4) existence of a dedicated authority or independent mental health body. Responses to these variables were present (Yes) or absent (No).

##### Resources for mental health

The variables extracted under resources for mental health were: (1) total number of mental health workers per 100,000 population; (2) number of psychiatrists; (3) number of mental health nurses; and (4) clinical psychologists per 100, 000 population. Responses to these variables were continuous.

##### Suicide prevention programs

Data were collected on whether the countries have suicide prevention programs, with Yes or No as the response options.

### Data analytic strategy

Data were summarized using descriptive statistics, notably frequencies, percentages, and bar chart. This was followed by inferential statistics using chi square (χ^2^) and binomial logistic regression, with alpha level set at .05. The principal outcome variable, national suicide rates, and the resources for mental health variables did not follow Gaussian distribution. Consequently, we proceeded with the inferential statistical analysis by dichotomizing these variables [31]. First, they were numerically equalized and scaled along a common standard metric by converting them into z-scores. The standardized scores were subsequently recoded such that scores below and above the mean were designated as low (0) and high (1), respectively. The Yes/No responses to the variables under the mental health system governance domain were also recoded as 1 and 0, respectively, for statistical purposes.

Next, χ^2^ analysis was conducted to determine the association between the independent variables and suicides. Phi and Cramer’s V coefficients were used to estimate the strength of the association, with coefficient values between .10 and .29, .30 to .49, and values at .50 and above interpreted as small, moderate and large effects. This was followed by a binomial logistic regression to determine the predictors of suicide categorization using odd ratios (OR) and adjusted odd ratio (AOR), where applicable. Income group and the variables under mental health system governance and resource for mental health domains were entered into the regression model independently. Only the variables demonstrating significant associations with suicide categorization based on χ^2^ analysis were included in this analysis. Last, because national income is a major determinant of several indicators of mental health, including the propensity to increase the number of mental health professionals and enact mental health legislations [5], the effect of income group was controlled for to obtain the ‘true effect’ of mental health legislation and professionals on suicide categorization. All analyses were performed using SPSS version 24.

## Results

### National income and suicide

The average suicide mortality rate for 155 countries was 8.96 (Range: .50–31.90). Income group-based analyses revealed that low income (*n* = 27), low-middle income (*n* = 42), upper-middle income (*n* = 46) and high-income countries (*n* = 40) recorded 7.06 (range: 3.7–12.20), 7.61 (range: 1.90–22.40), 8.49 (range: 1.70–31.00) and 12.18 (.50–31.90) average suicide mortality rate, respectively. Income group and suicide rate are significantly correlated, χ^2^ (3) = 14.47, *p* < .01, Phi & Cramer’s V = .31. Countries recording at least 20 suicide rate per 100, 000 include Belgium, Latvia, Ukraine, Suriname, South Korea, Guyana, Russian Federation, and Lithuania. In contrast, Antigua and Barbuda, Barbados, Grenada, Bahamas and Syrian Arab Republic were among the countries that reported less than two suicide rate per 100, 000. From Table 1, national income and suicide rate evinced statistically significant relationship, χ2 (3) = 14.47, p < .05. Further analyses showed that the odds of being designated as high suicide risk did not differ significantly between low income (the reference category) and low-middle and upper-middle income countries (*p* > .05). In contrast, high-income countries have higher odds of being classified as high suicide risk (*b* = 1.67, OR = 5.31).

**Table 1.** Relationship between national income, mental health system indicators and suicide

	Suicide				
Variables	Low, *n*(%)	High, *n*(%)	Total, *n*(%)	χ2	P/C
**National Income**				14.47**	.31
Low income	20(21.5)	7(11.3)	27(17.4)		
Low middle income	28(30.1)	14(22.6)	42(27.1)		
Upper middle income	31(33.3)	15(24.2)	46(29.7)		
High income	14(15.1)	26(41.9)	40(25.8)		
Total	93(60)	62(40)	155(100)		
**Mental Health Professionals**					
Total mental health professionals Low High Total	73(90.1) 8(9.9) 81(62.8)	28(58.3) 20(41.7) 48(37.2)	101(78.3) 28(21.7) 129(100)	17.92***	.37
Psychiatrists Low High Total	72(90) 8(10) 80(62)	21(42.9) 28(57.1) 49(38)	93(72.1) 36(27.9) 129(100)	33.57***	.51
Mental health nurses Low High Total	62(88.6) 8(11.4) 70(64.8)	19(50) 19(50) 38(35.2)	81(75) 27(25) 108(100)	19.54***	.43
Clinical psychologists Low High Total	65(94.2) 4(5.8) 69(65.7)	27(75) 9(25) 36(34.3)	92(87.6) 13(12.4) 105(100)	8.04**	.28
**Mental Health Governance System**					
Mental policies or plans No Yes Total	8(8.6) 85(91.4) 93(60)	6(9.7) 56(90.3) 62(40)	14(9) 141(91) 155(100)	.05	.02
Mental health law No Yes Total	35(37.6) 58(62.4) 93(60)	21(33.9) 41(66.1) 62(40)	56(36.1) 99(63.9) 155(100)	.04	.04
Child/adolescent mental health strategy No Yes Total	61(65.6) 32(34.4) 93(60)	27(43.5) 35(56.5) 62(40)	88(56.8) 67(43.2) 155(100)	7.37**	.22
Mental health authority/commission No Yes Total	53(57) 40(43) 93(60)	22(35.5) 40(64.5) 62(40)	75(48.4) 80(51.6) 155(100)	6.89**	.21
**Suicide prevention strategy**				10.57**	.26
No	75(80.6)	35(56.5)	110(71)		
Yes	18(19.4)	27(43.5)	45(29)		
Total	93(60)	62(40)	155(100)		

P/C = Phi and Cramer’s coefficient.

** = *p* < .01; *** = *p* < .001.

### Mental health professionals and suicide

The average number of mental health professionals per 100, 000 for 137 countries that supplied complete data was 37.61. When analyzed against income group, it was observed that the average number of mental health professionals differ across the income groups; low income (i.e. 1.58), lower-middle income (i.e. 7.33), upper-middle income (i.e. 43.61), and high income (i.e. 90.12) countries. Chi square analysis revealed that high-income countries are significantly likely to have more mental health professionals per 100,000, relative to low middle and low-income countries, χ^2^ (3) = 45.07, *p* < .001, Phi& Cramer’s V = .58. High-income countries with the highest number of mental health professionals include Finland (i.e. 250.55), USA (i.e. 271.28), Brazil (i.e. 317.45), Costa Rica (i.e. 341.94), and Monaco (i.e. 405.41). The least number of mental health professionals was recorded in sub-Saharan Africa (SSA) countries such as Chad (i.e. 0.04), Guinea (i.e. 0.05), Central African Republic (0.15), Mali (i.e. 0.16), and Kenya (i.e. 0.19). Similar trend was observed when the analysis was focused on specific mental health professionals, namely psychiatrists, mental health nurses and clinical psychologists.

As can be seen in Table 2, the odds of being designated as high risk for suicide is significantly higher among countries with a high number of mental health professionals (*b* = 1.88, OR = 6.52)., psychiatrists (*b* = 2.49, OR = 12.00), mental health nurses (*b* = 2.05, OR = 7.75) and clinical psychologists (*b* = 1.69, OR = 5.42). When the effect of national income was statistically controlled for, all the variables but clinical psychologists, retained their statistical significance (p. < .05).

**Table 2.** Logistic regression of suicide on income group and mental health indicators

Variables	*b*	95% CI Odd ratio (Unadjusted)	*B*	95% CI Adjusted Odd ratio
**Income Group**				
Low versus low-middle income	.36	1.43 (.49, 4.18)	-	-
Low versus upper-middle income	.32	1.38 (.48, 3.99)	-	-
Low versus high income	1.67**	5.31 (1.81, 3.60)	-	-
**Mental Health Personal (per 100,000)**				
Total mental health professionals	1.88***	6.52 (2.56, 5.49)	1.50**	4.49 (1.47, 4.73)
Psychiatrists	2.49***	12.00 (4.76, 6.23)	2.71***	15.04 (4.23, 7.41)
Nurses	2.05***	7.75 (2.93, 4.50)	1.31**	6.10 (1.93, 3.29)
Psychologists	1.69**	5.42 (1.54, 3.10)	1.25	3.50 (.90, 3.69)
**Mental health professionals**				
Child/adolescent strategy	.91**	2.47 (1.28, 4.78)	.68	1.98 (.97, 4.05)
Authority/Commission	.88**	2.41 (1.24, 4.67)	.73*	2.07 (1.01, 4.21)
**Suicide prevention programs**	1.17**	3.21(1.57, 6.60)	1.06**	2.87(1.31, 5.31)

** = *p* < .01; *** = *p* < .001.

### Mental health system governance and suicide

A total of 162 countries provided data on mental health system governance. A large proportion of the countries (*n* = 147, 90.7%) have instituted mental health policies or plans. More than half of the countries (*n* = 105, 64.8%) reported that they have stand-alone mental health laws, whereas 83 countries (51.2%) have a dedicated authority or independent mental health commission that oversees mental health activities in their respective countries. With respect to child/adolescent mental health, approximately 57% (*n* = 92) of the countries indicated they do not have plan or strategy for child/adolescent mental health. A statistically significant association was observed between income group and existence of plan or strategy for child/adolescent mental health, χ^2^ (3) = 17.23, *p* = .001, Phi & Cramer’s V = .33, dedicated mental health authority, χ^2^ (3) = 11.71, *p* = .008, Phi & Cramer’s V = .27, and stand-alone mental health law, χ^2^ (3) = 15.36, *p* = .002, Phi & Cramer’s V = .31, but not with mental health plans/policies χ^2^ (3) = 1.33, *p* = .722, Phi & Cramer’s V = .09.

Suicide rate correlated significantly with the availability of child/adolescent mental health strategies, χ^2^ (1) = 7.37, *p* < .01, Phi & Cramer’s V = .22, and the existence of independent mental health authority or commission, χ^2^ (1) = 6.89, *p* < .01, Phi& Cramer’s V = .21], but not with the availability of mental health plans/policies and mental health laws (*p* > .05). Further analyses reveal that, the odds of suicide rate is significantly higher in countries with child/adolescent mental health strategies (*b* = .91, OR = 2.47) and independent mental health authority (*b* = .88, OR = 2.41). When the effect of national income was controlled for, only the existence of independent mental health authority significantly increased the odds of being labelled as high risk for suicide (b = .73 AOR = 2.07).

### Suicide prevention programs and suicide

Of the 162 countries providing data on suicide, majority (*n* = 116, 71.6%) indicated they do not have stand-alone, government initiated national suicide prevention programs/plan. Income group correlated significantly with the existence of suicide prevention strategies, χ^2^ (3) = 15.34, p < .01, Phi & Cramer’s V = .31, with high-income group more likely to have national suicide prevention strategies. Indeed, when the analysis was disaggregated by income group, it was observed that only four low income (*n* = 27; i.e. Afghanistan, Mozambique, Chad and Uganda), five low-middle income (*n* = 43; e.g. Philippines, Vanuatu, Timor-Lesta, Bhutan and Nicaragua), 18 upper middle income (*n* = 49; e.g. Iran, Panama, Malaysia, Ecuador and Turkey) and 19 high-income countries (*n* = 43; e.g. Monaco, Israel, Italy and Spain) have national suicide prevention plans/strategies.

As shown in Table 1, availability of national suicide prevention plans was significantly associated with suicide rate, χ^2^ (1) = 10.57, *p* < .01, Phi & Cramer’s V = .26. Further analysis revealed that countries with suicide prevention strategies are significantly more likely to have higher odds of being categorized as high suicide risk country (*b* = 1.17, OR = 3.21), even after controlling for national income groupings (*b* = 1.06, AOR = 2.87).

## Discussion

The study primarily investigated the macro-level factors influencing suicide mortality rates across countries using data from the WHO’s 2017 Mental Health Atlas.

### Suicide rates in low and middle income and high-income countries

While suicide remains one of the global challenges confronting nations, the study found that the average suicide mortality rate was significantly higher in high-income countries, relative to low-income countries. This finding, which is largely consistent with previous studies [18,27,28], contradicts the widely held view that LMICs tend to experience the greatest proportion of suicides [3]. The supposedly low prevalence of suicide in LMIC could be accounted for by several factors, including the relatively high social support, religious commitment and/or involvement, and better family cohesion, which are potential protective factors against suicide [32,33].

The foregoing notwithstanding, there is also the possibility that real-suicide figures are obscured in LMIC partly due to the fundamental problem of inaccurate or lack of data pertaining to suicide. Indeed, suicide rates depend not only on the efficiency of civil registration systems, which are generally poor in LMIC, but also on the reporting of deaths which is in turn heavily influenced by the social, cultural and legal consequences of suicide [34]. Despite the ongoing campaign and advocacy to decriminalize suicide across countries, suicide continues to exist as a legal term that is often accompanied by legally prescribed punitive measures in several sub-Saharan African countries, including Ghana [35], Nigeria, Botswana, Gambia, Kenya, Malawi, Tanzania, Zambia and Ugandan [36]. In some LMICs such as Ghana, suicide is viewed as a taboo and unnatural death. Given this orientation, individuals expressing suicidal tendencies or persons deceased by suicides as well as their families are socially sanctioned. This observation incentivize families and communities to conceal or misreport suicidal behaviors so as to protect and preserve the sanctity of the family name [37,38]. Under-reporting of suicide is therefore highly prevalent in LMICs [28,39] owing to the prevailing social, cultural and legal proscriptions against suicides [5]. In contrast, the reported high prevalence of suicide in high-income countries could be due to an artifact of more efficient death registration systems and their case finding effects [34]. Likewise, the implementation of suicide prevention programs and the associated awareness and hypervilgilance could culminate into case-finding effect where there is the general tendency to label deaths as suicides [21].

The foregoing has enormous implication on the use of population suicide rate as a proxy indicator of the effectiveness of a country’s mental health services. That is, although LMIC reportedly have low suicide rates, it will be problematic to attribute this to better mental health system in these countries, as discussed previously. Indeed, LMICs are characterized by low number of mental health professionals, relative to high-income countries; a development that has been attributed to low investment in training mental health professionals and the relocation of mental health professionals to high-income countries for better conditions of service. For instance, on the latter point, an earlier study reported more Ghanaian psychiatrists working in the USA than those working in Ghana [40].

### Correlates of suicides rate

The study also found that high-income countries are significantly more likely to have child and adolescent mental health strategies, dedicated mental health authority and suicide prevention programs, relative to LMIC. More importantly, given the robust link between mental health problems and tendency for suicide particularly in the high-income countries [9,11], it is plausible to reason that programs and initiatives designed to promote and restore mental health functioning will contribute to a reduction in suicide rate. In contrast, however, the study found that macro-level mental health indicators examined in this study (e.g. number of mental health professionals, dedicated mental health authority and national suicide prevention programs) are associated with an increase in suicide rate, even after controlling for national income. This is largely consistent with previous studies [5,21]. Although the finding is somewhat counterintuitive and so could discourage further investment in mental health, on the other hand, it presents another opportunity for policy makers and relevant stakeholders to reexamine the various national mental health initiatives within the ambit of suicide prevention [21]. More specifically, the study highlighted several possibilities, including the view that the aforementioned national mental health initiatives may not be designed with a focus on suicide prevention, notwithstanding that they are intended to promote general mental health and wellbeing. Moreover, the over-medicalization of suicide, predominantly in high-income countries implies that greater attention is paid to urgency-driven curative medical solutions, thereby belittling the importance of associated sociocultural and economic factors [5,41]. Indeed, the observation that suicide can occur in the absence of psychological and mental health problems [42] implies that programs mainly designed to promote and improve mental health in general may have contributed little to reducing the prevalence of suicide rates. Some major risk factors that have been found to have exhibited direct and indirect relationship with suicide include unemployment, alcohol consumption and substance misuse in general, social inequalities, loss of social cohesion, and financial difficulties emanating from high indebtedness and bankruptcy [43–45]. An earlier study, for instance, found that the relationship between unemployment and suicide was statistically significant even after controlling for mental illness [46]. The foregoing presupposes that appropriate macroeconomic and social welfare policies that ensure and promote basic human rights, social security, gender equality and equitable development may contribute to reducing population suicide rates [5]. Perhaps, it is against this background that others have renewed calls for situating suicide research and prevention within social and cultural contexts [47]. The purpose is to deepen the understanding of suicides in accordance with postulation of the stress-diathesis model suggesting that suicide risk is multi-factorial [48] and should therefore not be reduced to only psychiatric illness [6]. Furthermore, although specific suicide prevention programs may exist particularly in high-income countries, there is the possibility they have not been implemented [49], or even if they are implemented, they could be riddled with implementation issues that could thwart or reduce their effectiveness.

## Limitations

The findings of the study should be reviewed in light of the following limitations. First, all ecological studies have the potential limitation of ecological fallacy, which can occur as an association observed between the study variables on the aggregate level will not necessarily represent the association existing at the individual level. Causal associations cannot be assumed since this study analyzed data collected using a cross-sectional study design. The study could also be confounded by potential variables such as cultural and religious differences between countries which were not captured in the WHO’s 2017 Mental Health Atlas. There is also the possibility of under or over-reporting of data which may occur for several reasons, including an attempt to present a good picture of mental health situations. The application of advanced data mining techniques may uncover patterns that might have been missed with the data analytic strategy employed in this study. Also, because the quality of training and focus of psychiatrists might differ across countries [22], the lack of data on these critical variables could affect the findings of the study. Last, the findings reported in this study could have been different with data from all WHO member countries. Notwithstanding the limitations, the findings reported in this study are largely consistent with previous ecological studies.

## Conclusion

This ecological study examined the association between population suicide rates and key mental health indicators based on the most recent WHO Mental Health Atlas which reported suicide rates from 191 countries. Findings generally revealed significant and positive relationship between macro-level mental health indicators and suicides. Although the findings appear somewhat counterintuitive in the light of the robust link between suicide and mental illnesses in many countries, nonetheless, they prompt action towards improving suicide reporting and recording systems across the globe generally and mapping of locally relevant factors contributing to suicides as a guide for formulating and implementing context sensitive prevention and intervention measures. .

Noteworthy is the lower rate of suicides in individual countries within the LMICs, which is as revealing as it is intriguing. Suicide and suicide prevention within most LMIC countries are not national priorities [50]. Apart from the social, cultural and legal barriers that hinder accurate reporting and recording of suicides, it is possible that the burden of disease attributable to communicable and infectious diseases in LMICs tended to sway concerted national health responses away from non-communicable and relatively ‘rare’ health problems such as suicides. Where modest attention was given to the latter, it was largely focused on cardiovascular diseases, diabetes, cancer and chronic pulmonary diseases [51,52]. However, given that some countries in the LMICs have or are undergoing ‘epidemiological transition’ whereby the relative importance of infectious diseases is gradually becoming less than that of chronic diseases [53], there is a need for national governments within the LMICs to commit more resources to non-infectious causes of mortality and morbidity such as suicides. Such efforts will be in keeping with the target 3.4 of the Sustainable Development Goals, which is to reduce by one-third premature mortality from non-communicable diseases by the year 2030 [54]. In the light of the above, we suggest the need to increase qualitative and quantitative research within individual countries that will help deepen understanding of the meaning of suicide and identifiable risk factors for people and how and why common risk factors contribute to suicides in some people but not in others.

## Paper context

Despite the robust association between mental health status and risk for suicide at the individual level, ecological studies exploring mental health systems and suicide rates are not only scarce but have produced inconsistent results. The study found that (high income) countries with proper mental health system and governance are more likely to record high suicide rates. There is the need to make existing mental health systems very responsive to suicide prevention.
